# Hydrogen Sulfide in Hypertension and Kidney Disease of Developmental Origins

**DOI:** 10.3390/ijms19051438

**Published:** 2018-05-11

**Authors:** Chien-Ning Hsu, You-Lin Tain

**Affiliations:** 1 Department of Pharmacy, Kaohsiung Chang Gung Memorial Hospital, Kaohsiung 833, Taiwan; chien_ning_hsu@hotmail.com; 2 Departments of Pediatrics, Kaohsiung Chang Gung Memorial Hospital and Chang Gung University College of Medicine, Kaohsiung 833, Taiwan; 3 Institute for Translational Research in Biomedicine, Kaohsiung Chang Gung Memorial Hospital and Chang Gung University College of Medicine, Kaohsiung 833, Taiwan

**Keywords:** developmental origins of health and disease (DOHaD), hydrogen sulfide, hypertension, kidney disease, nitric oxide, nutrient-sensing signals, oxidative stress, renin–angiotensin system

## Abstract

Adverse environments occurring during kidney development may produce long-term programming effects, namely renal programming, to create increased vulnerability to the development of later-life hypertension and kidney disease. Conversely, reprogramming is a strategy aimed at reversing the programming processes in early life, even before the onset of clinical symptoms, which may counter the rising epidemic of hypertension and kidney disease. Hydrogen sulfide (H_2_S), the third gasotransmitter, plays a key role in blood pressure regulation and renal physiology. This review will first present the role of H_2_S in the renal system and provide evidence for the links between H_2_S signaling and the underlying mechanisms of renal programming, including the renin–angiotensin system, oxidative stress, nutrient-sensing signals, sodium transporters, and epigenetic regulation. This will be followed by potential H_2_S treatment modalities that may serve as reprogramming strategies to prevent hypertension and kidney disease of developmental origins. These H_2_S treatment modalities include precursors for H_2_S synthesis, H_2_S donors, and natural plant-derived compounds. Despite emerging evidence from experimental studies in support of reprogramming strategies targeting the H_2_S signaling pathway to protect against hypertension and kidney disease of developmental origins, these results need further clinical translation.

## 1. DOHaD Concept: Hypertension and Kidney Disease of Developmental Origins

One in three adults worldwide has high blood pressure (BP). The prevalence of hypertension is even higher among patients with kidney disease. Conversely, kidney disease is the most common form of secondary hypertension. In addition to sharing many common risk factors, hypertension and kidney disease could be the cause and consequence of each other. Hypertension as well as kidney disease, when found early, can be treated to prevent more related disorders and serious complications. Environmental factors in fetal and infantile life can be important early origins of adult disorders due to fetal programming that permanently shapes the body’s morphology and function and contributes to adult disease. This idea is framed as the developmental origins of health and disease (DOHaD) [1]. Notably, following the concept of DOHaD, both of these disorders may originate from adverse early-life environmental insults [2,3,4]. 

Kidneys play a decisive role in blood pressure (BP) regulation. Adverse environments occurring during nephrogenesis may produce long-term programming effects on the structure and/or function of the kidney, formally known as renal programming [5], which may increase the vulnerability to developing hypertension and kidney disease in adulthood. Conversely, reprogramming, a strategy aimed at reversing the initial programming processes, is afforded by the DOHaD concept to shift therapeutic interventions from adulthood to early life, even before the onset of clinical symptoms [6]. A growing body of evidence suggests that hydrogen sulfide (H_2_S) plays a crucial role in the normal physiology and pathogenesis of many disorders, and many therapeutic targets exist for H_2_S therapy [7,8]. Based on the two aspects of the DOHaD concept, this review will first present the evidence for the programming mechanisms that may lead to hypertension and kidney disease of developmental origins, with a focus on the kidney. This will be followed by potential pharmacological interventions targeting H_2_S signaling that may serve as a reprogramming strategy to counter the growing epidemic of hypertension and kidney disease.

## 2. Hydrogen Sulfide in Renal System

### 2.1. H_2_S Production and Biological Function

**Figure 1:**
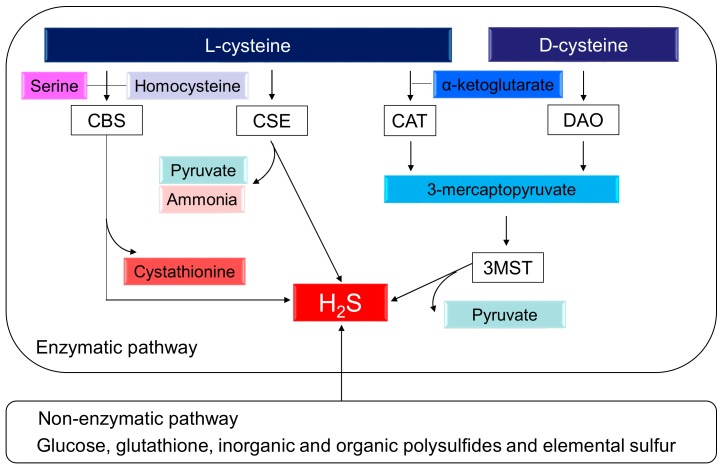
Schematic representation of enzymatic and nonenzymatic H_2_S synthesis pathways. Cystathionine β-synthase (CBS) catalyzes homocysteine and serine to produce H_2_S and l-cystathionine. Cystathionine γ-lyase (CSE) catalyzes l-cysteine to generate H_2_S, pyruvate, and ammonia. 3-Mercaptopyruvate sulfurtransferase (3MST) produces H_2_S from 3-mercaptopyruvate (3MP), which is generated by cysteine aminotransferase (CAT) and d-amino acid oxidase (DAO) from l-cysteine and d-cysteine, respectively. In addition to enzymatic synthesis pathways, endogenous production of H_2_S can also occur through other nonenzymatic processes.

H_2_S, the third gasotransmitter, next to nitric oxide (NO) and carbon monoxide (CO), plays a key role in BP control and renal physiology [7,8,9]. The production of H_2_S can occur via two pathways, enzymatic and nonenzymatic. Enzymatic synthesis of H_2_S from l-cysteine occurs through three enzymes, cystathionine β-synthase (CBS), cystathionine γ-lyase (CSE), and 3-mercaptopyruvate sulphurtransferase (3MST), which work together with cysteine amino transferase (CAT) [7]. l-cysteine serves as a substrate for both CBS and CSE to form H_2_S. CBS and CSE can generate H_2_S using many different substrates. CBS catalyzes homocysteine and serine to generate cystathionine. CSE catalyzes l-cysteine to produce pyruvate, H_2_S, and ammonia. CSE also breaks down cystathionine into l-cysteine, α-ketobutyrate, and ammonia. In an alternative pathway, CAT and D-amino acid oxidase (DAO) provide 3-mercaptopyruvate (3MP) for 3MST to produce H_2_S. Additionally, d-cysteine can be metabolized by DAO to generate H_2_S in the kidney [10]. In the presence of α-ketoglutarate, CAT metabolizes l-cysteine to form 3MP, whereas DAO produces 3MP from d-cysteine. All of these H_2_S-generating enzymes are expressed abundantly in the kidney. Interestingly, renal production of H_2_S from d-cysteine is approximately 80 times greater than that from l-cysteine [10]. Alternatively, nonenzymatic production of H_2_S occurs through glucose, glutathione, inorganic and organic polysulfides, and elemental sulfur. The biochemical pathways related to the H_2_S synthesis are illustrated in Figure 1. 

H_2_S has diverse and widespread biological functions, including vasodilatation, mitochondria bioenergetics, metabolic modulation, renal excretory function, angiogenesis, anti-inflammation, antioxidant, and antiapoptosis [7,8,9]. Until recently, the biological mechanisms of H_2_S signaling have been incompletely understood. One pathway for H_2_S signaling is through protein sulfhydration by the formation of a persulfide bond to the reactive cysteine residues of target proteins [11]. There is some evidence that H_2_S signaling regulates physiological processes via sulfhydration, but the mechanisms involved are still not well characterized. 

### 2.2. Antihypertensive and Renoprotective Effects of H_2_S

In the spontaneously hypertensive rat (SHR), a commonly used genetic hypertensive model, H_2_S deficiency appears prior to the development of hypertension, whereas the administration of exogenous H_2_S donor (NaHS) protects SHRs against hypertension [12]. The contribution of H_2_S deficiency in hypertension has been studied in other models of hypertension, including the renovascular hypertensive model [13], NO-deficient rats [14], and Dahl salt-sensitive rats [15]. In agreement with these studies, CSE knockout mice have also been found to have reduced levels of H_2_S and hypertension [16]. However, this could not be confirmed in another type of CSE knockout mouse model on a different genetic background [17]. H_2_S signaling deficiencies have also been associated with various kidney diseases, such as renal ischemia/reperfusion injury, obstructive nephropathy, diabetic nephropathy, hypertensive nephropathy, and the 5/6 nephrectomy rat model of chronic kidney disease. These models have been reviewed elsewhere [18,19]. Conversely, a number of recent studies stress the protective role of exogenous and endogenous H_2_S on hypertension and kidney disease, and this has also been reviewed elsewhere [20,21,22]. However, limited information is available on whether these beneficial mechanisms could be programmed in response to early-life insults in the kidney, to reduce the vulnerability to developing later hypertension and kidney disease.

## 3. Common Mechanisms Link H_2_S to Renal Programming

Though a variety of organ systems can be programmed in response to early-life adverse environmental insults, renal programming is considered crucial in the development of hypertension and kidney disease [4,5,6]. Despite the diversity of early-life insults, emerging evidence indicates that there may be common mechanisms underlying renal programming which lead to the pathogenesis of hypertension and kidney disease of developmental origins. Though the common pathogenic mechanisms are still inconclusive, animal models have provided insight on particular pathways including the renin–angiotensin system (RAS), oxidative stress, nutrient-sensing signals, dysregulation of sodium transporters, and epigenetic regulation [2,3,4,5,6,23]. Notably, these extensive experimental animal studies have demonstrated interplay between H_2_S and the abovementioned mechanisms. Each of these examples will be discussed in turn.

### 3.1. Renin–Angiotensin System

The RAS is a well-known hormonal cascade controlling BP and kidney development [24,25]. Currently, there are two main axes of the RAS: (1) the angiotensin-converting enzyme (ACE)–angiotensin (Ang) II–angiotensin type 1 receptor (AT1R) classical axis and (2) the ACE2–angiotensin (1–7)–Mas receptor axis [24]. Both axes have been examined in relation to their impacts on fetal programming [26,27]. There is a biphasic response with reduced classical RAS expression at birth, which becomes normalized with age [5]. Under pathophysiological scenarios where this normalization overcompensates, early-life renal programming subsequently activates the classical RAS, resulting in hypertension and kidney disease development in later life [5]. Conversely, early blockade of the classical RAS has been shown to prevent the development of hypertension [28,29,30]. These observations support the view that RAS may be an underlying mechanism involved in renal programming and the development of hypertension and kidney disease. Decreased H_2_S levels or downregulation of H_2_S-generating enzymes have been found in some hypertensive models with activation of the RAS [31,32]. Exogeneous H_2_S administration has been shown to suppress renin release [32], downregulate renal mRNA expression of *Ren*, *Atp6ap2*, *Agt*, *Ace*, and *Agtr1a* [33], and decrease protein expression of AT1R [34], resulting in protection from hypertension. Nevertheless, the detailed mechanisms underlying the modulation of RAS by H_2_S contributing to the protection of programmed kidney disease need to be further investigated. 

### 3.2. Oxidative Stress

The development of the embryo occurs in a relatively low-oxygen environment, and the developing fetus is highly vulnerable to oxidant injury due to its low antioxidant capacity [35]. Oxidative stress is an oxidative shift characterized by an imbalance between oxidants (e.g., reactive oxygen species (ROS) or reactive nitrogen species (RNS)) and antioxidants in favor of oxidants. A number of recent studies support the importance of oxidative stress in relation to programmed hypertension and kidney disease in different models, including pre-eclampsia, caloric restriction, maternal diabetes, prenatal dexamethasone exposure, high fructose intake, maternal smoking, and low-protein diet [6,36]. On the contrary, some reprogramming strategies have aimed at influencing the balance of NO–ROS to reduce oxidative stress and thus prevent hypertension and kidney disease of developmental origins [36]. H_2_S is a Janus-faced molecule that acts either as a pro-oxidant or antioxidant [37]. It can interact with ROS or RNS to generate free radicals. Conversely, H_2_S has an antioxidant property, by which it modulates the concentration of glutathione, an important intracellular thiolic antioxidant, and acts as a free radical scavenger [38]. Like NO, H_2_S causes vasodilatation and there is crosstalk between these two molecules to regulate BP [7,8]. Thus, these findings suggest that suppression of oxidative stress may contribute to antihypertensive effects of H_2_S. However, whether the antioxidative ability of H_2_S in itself is important in reducing the BP in concert with other BP-lowering actions remain to be clarified.

### 3.3. Nutrient-Sensing Signals

Imbalanced maternal nutrition and metabolic insults can disturb nutrient-sensing signals, which play a key role in fetal metabolism and development, resulting in renal programming and developmental hypertension [39,40]. A number of nutrient-sensing signaling pathways exist in the kidney, including cyclic adenosine monophosphate (AMP)-activated protein kinase (AMPK), silent information regulator transcript (SIRT), peroxisome proliferator-activated receptors (PPARs), and PPARγ coactivator-1α (PGC-1α) [41]. Early-life nutritional insults can drive nutrient-sensing signals (e.g., AMPK, SIRT1, and PGC-1α) to regulate PPARs and their target genes, thereby promoting programmed hypertension and kidney disease [42]. AMPK, SIRT1, and PGC-1α can also promote autophagy, a lysosome-mediated degradation process for damaged cellular constituents. Since dysregulation of autophagy causes abnormal mitochondrial function and increased oxidative stress [43], early interventions by AMPK activators or PPAR modulators have been examined as reprogramming strategies in DOHaD research [42,44]. It is noteworthy that one of the beneficial effects of H_2_S is the activation of AMPK and promotion of autophagy [45]. Further efforts are required to advance our understanding of the long-term programming effects of H_2_S on nutrient-sensing signals and their relationships to hypertension and developmental kidney disease.

### 3.4. Sodium Transporters

Fetal programming induced by early-life environmental insults that contribute to the development of hypertension is related to enhanced sodium reabsorption attributed to increased expression of sodium transporters [4]. These insults include prenatal glucocorticoid exposure [46,47], maternal low-protein diet [48], maternal high-fat diet [49], and maternal exposure to continuous light [50]. Several sodium transporters have been identified in these processes, including the Na-K-2Cl cotransporter (NKCC2), Na^+^/Cl^−^ cotransporter (NCC), type 3 sodium hydrogen exchanger (NHE3), and the Na^+^/K^+^ATPase α1 subunit (NaKATPase). In addition to prenatal insults, our previous study has shown that a maternal high-fructose diet plus postnatal high-salt diet increased renal levels of NKCC2, NHE3, and NCC in a two-hit model of programmed hypertension [51]. Therefore, regardless of whether the insults are prenatal or postnatal, the dysregulation of sodium transporters resulting in inappropriate sodium reabsorption could increase the vulnerability to adult hypertension and kidney disease. Notably, an exogenous H_2_S donor (NaHS) has been reported to inhibit expression and/or activity of several sodium transporters, which may account for its protective effects on renal function and hypertension [52,53]. Nevertheless, there remains a lack of definitive data on how H_2_S affects sodium transporters from animal models of programmed hypertension and kidney disease. It will be of great value to reveal the reprogramming effect of H_2_S on sodium transporters in different models of programmed hypertension and kidney disease.

### 3.5. Epigenetic Regulation

Epigenetic regulation such as histone modifications, DNA methylation, and noncoding RNAs are involved in mediating the effects of early-life environmental influences on later-life health [54]. Epigenetics refers to alterations in gene expression that are not explained by changes in the DNA sequence. Until recently, little was known about the role of epigenetics in renal programming. A previous report has shown that maternal folic acid supplementation did not alter global DNA methylation in the offspring’s kidney [55]. Another study demonstrated that renal microRNA altered the protein restriction model of fetal programming [56]. Additionally, only a few studies using high-throughput DNA sequencing technologies report that different early-life insults permanently alter the renal transcriptome expression profile in rat offspring [40,57]. Conversely, emerging evidence support the idea that H_2_S has epigenetic effects by mediating DNA methylation [34], histone deacetylase (HDAC) [58], and microRNA expression [59]. These findings suggest that epigenetic regulation may be an important link between H_2_S signaling and renal programming.

**Figure 2:**
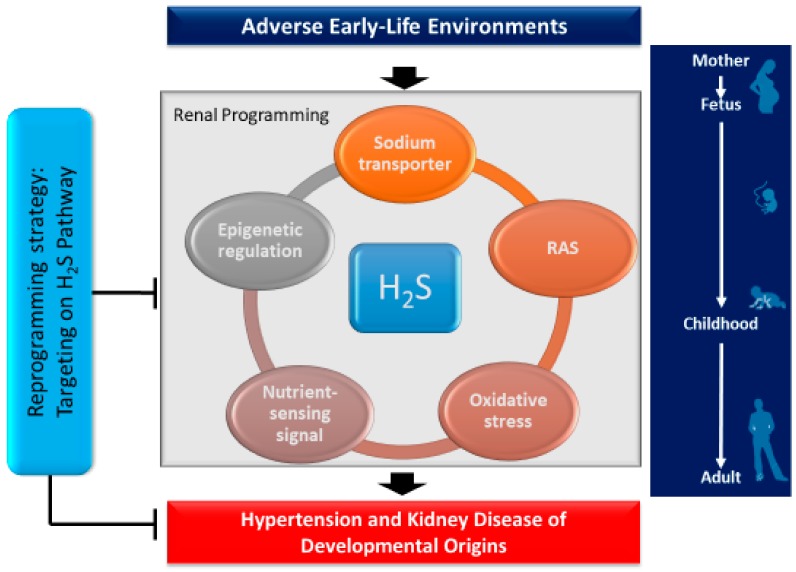
Schema outlining the potential role of H_2_S on mediating other mechanisms in the kidney leading to hypertension and developmental kidney disease in response to a variety of early-life insults. Targeting the H_2_S pathway could be a reprogramming strategy for programmed hypertension and kidney disease to reduce the lifetime burden by early intervention.

All of these observations demonstrate a close link between H_2_S and other important mechanisms involved in renal programming (Figure 2). Despite emerging evidence indicating crosstalk between H_2_S and particular mechanisms underlying programmed processes, we are still far from the conclusion that H_2_S may play a central role in mediating other mechanisms leading to hypertension and developmental kidney disease.

## 4. Reprogramming Strategy Targeting the H_2_S Signaling Pathway

Although excess H_2_S may contribute to some diseases, the application of H_2_S treatment modalities to increase H_2_S signaling attract the most attention [60]. As mentioned earlier, the protective effects of H_2_S signaling have been demonstrated in many disorders, including hypertension and kidney disease. Therefore, the H_2_S treatments presented here focus on approaches aimed to increase H_2_S signaling. Currently, available H_2_S treatment modalities include precursors for H_2_S synthesis, H_2_S donors, and natural plant-derived compounds. l-cysteine, N-acetylcysteine (NAC), and D-cysteine are precursors for endogenous H_2_S synthesis. H_2_S donors, including donor molecules and H_2_S-releasing drugs, can be divided into three types: inorganic sulfide salts (e.g., NaHS), organic compounds (e.g., GYY4137), and agonists of H_2_S-synthesized enzymes [60,61]. Additionally, garlic-derived polysulfides are considered to be an H_2_S treatment modality as they can stimulate the production of H_2_S [61].

Here, we summarize studies which document reprogramming in animal models of hypertension and kidney disease, focusing on interventions aimed at the H_2_S signaling pathway. Of note, pharmacotherapies will be narrowly restricted to key periods during early development. These H_2_S treatment modalities are listed in Table 1 [62,63,64,65,66,67,68]. This list is by no means complete and is expected to grow rapidly as the field of DOHaD research flourishes.

### 4.1. Precursors for H_2_S Synthesis

l-cysteine supplementation is widely used to create endogenous H_2_S in experimental studies. l-cysteine has been known to lower BP directly or through its storage form, glutathione, by decreasing oxidative stress, improving insulin resistance and glucose metabolism, and modulating NO [68]. N-acetylcysteine (NAC), a stable cysteine analogue, has been reported to prevent hypertension in experimental and human studies [68,69], which supports this idea. However, l-cysteine is not a good H_2_S precursor due to its multiple metabolic fates. Indeed, the reported antihypertensive and renoprotective effects may not be directly related to H_2_S signaling. Another H_2_S precursor, d-cysteine, is nutritionally antagonistic [70]; therefore, the antihypertensive effect of d-cysteine supplementation has received less attention. There is only one report demonstrating that d-cysteine supplementation protects the mouse kidney against ischemia/reperfusion injury [71].

So far, very few studies have addressed DOHaD research to explore the protective role of H_2_S precursors in the kidney in response to a variety of early-life insults. We first showed that early supplementation of d- or l-cysteine between 4 and 6 weeks of age (i.e., prehypertensive stage) can protect SHRs against high-salt-enhanced elevation of BP and kidney injury [62]. Although a previous report has shown that the d-cysteine pathway is 80-fold greater in H_2_S-producing activity in the kidney compared to the l-cysteine pathway [71], our data revealed that the protective effects on BP are similar. Correspondingly, early supplementation of NAC starting at four weeks of age has been reported to prevent hypertension in SHRs [63]. As such, the reprogramming effects of early NAC therapy on the protection of programmed hypertension have been reported in several animal models, including prenatal dexamethasone and postnatal high-fat diet [64], suramin-induced pre-eclampsia [65], N^G^-nitro-l-arginine-methyl-ester (L-NAME)-induced pre-eclampsia [66], and maternal nicotine exposure [67]. Despite the reprogramming effects of several H_2_S precursors in response to early-life insults that have been studied in experimental animal models, there remains a lack of data regarding their clinical translation. 

### 4.2. H_2_S Donors

In most studies, inorganic sulfide salts such as sodium hydrosulfide (NaHS) and sodium sulfide (Na_2_S) are used as H_2_S donors to generate exogenous H_2_S [60]. NaHS has been reported to be protective in various hypertensive models, including SHR [72], Ang II-infused mice [31], NO-deficient rats [14], and Dahl salt-sensitive rats [15]. Additionally, the protective effects of exogenous H_2_S derived from inorganic sulfide salts in different experimental models of kidney disease have been reviewed elsewhere [21,22]. However, inorganic sulfide salts induce a rapid but short-lived increase of H_2_S to supraphysiological concentrations [73]. To overcome this limitation, several organic slow-releasing H_2_S compounds have been synthesized [74]. Among them, GYY4137 showed a BP-lowering effect in both CSE inhibition-induced pre-eclampsia and L-NAME-treated SHR [75,76]. Additionally, GYY4137 caused both antihypertensive and renoprotective effects in an Ang II-induced hypertensive model [59]. In addition to slow-releasing H_2_S compounds, hybrids of H_2_S and other drugs are receiving attention. Although H_2_S-releasing hybrids such as SG1002 (H_2_S-NO hybrid) and ATB-346 (H_2_S-NSAID hybrid) are now being demonstrated in clinical trials, there remains a need to better understand the underlying mechanisms for the treatment of a wide range of disorders, especially hypertension and kidney disease [77]. 

In contrast to the multitude of studies in adults, there are very few DOHaD studies on the role of H_2_S donors in hypertension and developmental kidney disease. NaHS therapy between four and six weeks of age has been reported to prevent hypertension in adult SHRs [33]. Another report showed that renovascular hypertensive mother rats treated with NaHS can protect their offspring against hypertension [34]. However, none of the organic slow-releasing H_2_S compounds and H_2_S-releasing hybrids have been examined in terms of their beneficial effects on programmed hypertension and kidney disease.

### 4.3. Natural Plant-Derived Compounds

Garlic-derived polysulfide compounds have drawn attention as natural precursors of H_2_S. A growing body of reports have shown that garlic-derived polysulfide compounds have the potential for ameliorating hypertension and associated morbidity [78,79]. Polysulfides in garlic may also influence the regulation of the NO signaling pathway, leading to NO-mediated vasodilation and reduction of BP [79]. Also, garlic-derived compounds provide renoprotection in several models of kidney disease, such as renal ischemia/reperfusion injury [80], acetaminophen-induced nephrotoxicity [81], and gentamicin-induced nephrotoxicity [82]. However, the reprogramming effects of these compounds on hypertension and kidney disease of developmental origins have not yet been examined. 

### 4.4. Others

A number of currently used drugs have been reported to increase H_2_S concentrations, such as ramipril, atorvastatin, amlodipine, vitamin D, digoxin, aspirin, paracetamol, carvediol, testosterone, 17β-estradiol, and metformin [60]. Despite progress made in recent years on pharmacotherapies in the H_2_S field, very few studies have targeted their potential in reprogramming. With a better understanding on the interplay between the H_2_S signaling pathway and other mechanisms underlying renal programming, the application of reprogramming strategies targeting the abovementioned mechanisms is feasible for early intervention. 

For example, there is a growing body of evidence that AMPK activators possess therapeutic potential in treating hypertension and kidney disease [83,84]. Since AMPK is part of the nutrient-sensing signaling involved in renal programming, and AMPK is an important H_2_S downstream signal [45], it would be interesting to see whether AMPK activators may be a useful reprogramming strategy to prevent developmental programming of hypertension and kidney disease. Given the fact that most available H_2_S-releasing drugs are still in preclinical experiments, targeting the H_2_S downstream signal-related mechanisms would appear to be a practical approach for further clinical translation.

## 5. Conclusions

Advances in experimental research indicate that the H_2_S signaling pathway may provide potential therapeutic targets for treating hypertension and kidney disease. Targeting the H_2_S signaling pathway as a reprogramming strategy against hypertension and developmental kidney disease is a flourishing field and will become even more important in light of the growing epidemic of hypertension and kidney disease. 

This review has provided an overview on the various reprogramming strategies that are relevant to the H_2_S signaling pathway, including precursors for H_2_S synthesis, H_2_S donors, and natural plant-derived compounds. Also, reprogramming interventions may be achieved by targeting the H_2_S downstream signal, which mediates particular mechanisms of renal programming. Although emerging evidence from animal studies supports H_2_S as a reprogramming strategy for long-term protection against hypertension and developmental kidney disease, these results await further clinical translation.

## Figures and Tables

**Table 1:** Reprogramming strategy targeted on H_2_S signaling in models of programmed hypertension and kidney disease.

Animal Models	Gender/Species	Age at Evaluation	Dose and Period of Treatment	Reprogramming Effects	Ref.
Precursors for endogenous H_2_S synthesis					
High-salt SHR^1^	Male SHR	12 weeks	l-cysteine (8 mmol kg^−1^ body weight/day) from 4 to 6 weeks of age	Prevented hypertension; prevented renal injury	[62]
High-salt SHR	Male SHR	12 weeks	d-cysteine (8 mmol kg^−1^ body weight/day) from 4 to 6 weeks of age	Prevented hypertension; prevented renal injury	[62]
SHR	Male SHR	12 weeks	2% NAC^2^ in drinking water from 4 to 12 weeks of age	Prevented hypertension	[63]
Prenatal dexamethasone and postnatal high-fat diet	Male SD^3^ rats	12 weeks	1% NAC in drinking water during pregnancy and lactation	Prevented hypertension	[64]
Suramin-induced pre-eclampsia	Male SD rats	12 weeks	1% NAC in drinking water during pregnancy and lactation	Prevented hypertension	[65]
N^G^-nitro-l-arginine-methyl ester (l-NAME)-induced pre-eclampsia	Male SD rats	12 weeks	1% NAC in drinking water during pregnancy and lactation	Prevented hypertension	[66]
Maternal nicotine exposure	Male SD rats	8 months	NAC (500 mg/kg/day) in drinking water from gestational day 4 to postnatal day 10	Prevented hypertension	[67]
H_2_S donors					
SHR	Male SHR	12 weeks	NaHS (14 μmol/kg/day) daily intraperitoneal injection from 4 to 8 weeks of age	Prevented hypertension	[33]

^1^ SHR = spontaneously hypertensive rat; ^2^ NAC = N-acetylcysteine; ^3^ SD rats = Sprague–Dawley rats.

## Abbreviations

ACE	Angiotensin-converting enzyme
AMPK	Cyclic adenosine monophosphate (AMP)-activated protein kinase
AT1R	Angiotensin II type 1 receptor
CAT	Cysteine aminotransferase
CBS	Cystathionine β-synthase
CSE	Cystathionine γ-lyase
DAO	D-amino acid oxidase
DOHaD	Developmental origins of health and disease
L-NAME	N^G^-nitro-l-arginine-methyl-ester
NAC	N-acetylcysteine
NaHS	Sodium hydrosulfide
NCC	Na^+^/Cl^−^ cotransporter
NHE3	Type 3 sodium hydrogen exchanger
NKCC2	Na-K-2Cl cotransporter
PGC-1α	Peroxisome proliferator-activated receptor γ coactivator-1α
PPAR	Peroxisome proliferator-activated receptor
RAS	Renin–angiotensin system
SHR	Spontaneously hypertensive rat
SIRT	Silent information regulator transcript
3MST	3-mercaptopyruvate sulphurtransferase
